# Sustainable Bifunctional Electrospun Hybrid Nanofibers for CO_2_ Capture and Conversion

**DOI:** 10.1002/marc.202500050

**Published:** 2025-05-27

**Authors:** R. Hengsbach, I. Bychko, S. Schwarz, P. Strizhak, A. Fahmi

**Affiliations:** ^1^ Faculty of Technology and Bionics Rhine‐Waal University of Applied Science Marie‐Curie‐Straße 1 47533 Kleve Germany; ^2^ L. V. Pisarzhevskii Institute of Physical Chemistry National Academy of Sciences of Ukraine 31 Prosp. Nauky Kyiv 03028 Ukraine; ^3^ Leibniz‐Institut für Polymerforschung Dresden e. V. Hohe Straße 6 01069 Dresden Germany

**Keywords:** carbon capture, carbon conversion, hybrid systems, nanofibers, side‐by‐side electrospinning

## Abstract

Bifunctional nanofibers for CO_2_ capture and conversion can be fabricated by electrospinning. Using advanced methods like side‐by‐side electrospinning enables the integration of multiple independent functionalities. The combination of poly(ethylene oxide) (PEO) modified with poly(ethylene imine) (PEI) for CO_2_ capture and PEO loaded with copper nanoparticles (CuNP) for CO_2_ catalysis results in bifunctional fibers that can be synthesized using water as a green solvent. The fibers are characterized using scanning electron microscopy, thermogravimetric analysis, and differential scanning calorimetry. The bifunctional properties of fibers are illustrated by gas adsorption and catalytic experiments. The production via side‐by‐side electrospinning leads to materials with orthogonal properties that can be adjusted and optimized independently. The introduced imine groups capture CO_2_, which can be directly converted to methanol by hydrogenation at CuNP at a low temperature of 150 °C.

## Graphical abstract

Fabrication of bifunctional hybrid nanofibers is shown via side‐by‐side electrospinning. One part of the fibers contains amines for CO_2_ capturing and the other part, containing copper nanoparticles catalyzes CO_2_ hydrogenation to methanol at low temperatures of 150 °C. Both parts of the fibers are fabricated as single fibers and their characteristics are compared to the bifunctional hybrid nanofibers.

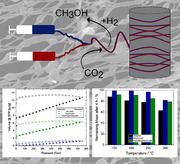

## 1 Introduction

The rising level of CO_2_ emissions and the resulting increase in atmospheric CO_2_ concentration demands urgent development of carbon capture and storage (CCS) or carbon capture and utilization (CCU) technologies.^[^ ^1^, ^2^, ^3^, ^4^ ^]^ These technologies are highly needed to follow the Paris Agreement of keeping global warming below 2 °C and even trying to keep it below 1.5 °C.^[^ ^2^ ^]^

Capturing of CO_2_ can be efficiently performed with amines.^[^ ^1^, ^3^, ^5^ ^]^ The utilization of captured CO_2_ is crucial for making it reusable and advancing toward a circular economy, where the obtained products can be used as chemical industry feedstocks or fuels.^[^ ^4^, ^6^, ^7^, ^8^ ^]^ One system combining capture and conversion properties reduces the energy input since compression and release of CO_2_ would not be necessary.^[^ ^9^, ^10^, ^11^ ^]^ Additionally, there is a thermodynamical advantage since the native state of gaseous CO_2_ will be altered by adsorption. CO_2_ is a thermodynamically and kinetically stable sp hybridized molecule. By adsorption, the linear structure of the molecule will be bending, and the formed trigonal planar sp^2^ anionic carboxylate is more favorable for further reactions.^[^ ^4^ ^]^ Dang et al. summarized different pathways for synthesizing methanol from CO_2_ hydrogenation.^[^ ^12^ ^]^ Otto et al. analyzed possible reaction pathways for bulk and fine chemicals.^[^ ^13^ ^]^

The production of nanofibers can be done by electrospinning on an industrial scale.^[^ ^14^, ^15^ ^]^ Electrospinning needs a spinneret, a metal needle, a polymer solution in a syringe that will be pumped evenly, and a collector. A high voltage is applied at the spinneret and the collector. The distance between them can be adjusted affecting the fiber's morphology. Caused by the high voltage stretching of the polymer jet will take place and a Taylor cone will form. The solvent evaporates after the solution leaves the spinneret forming the nanofibers.^[^ ^16^, ^17^ ^]^ Electrospinning is considered as a powerful tool for the synthesis of heterogenous catalysts,^[^ ^18^, ^19^ ^]^ CCU and CCS applications.^[^ ^20^ ^]^

Capturing of CO_2_ was already shown using nitrogen‐containing porous carbon fibers which were synthesized by electrospinning poly(acrylonitrile).^[^ ^21^ ^]^ Hong et al. recently reviewed the progress in using electrospun membranes for CO₂ capture, highlighting their growing importance due to their tunable morphology, controllable pore structure, high specific surface area, wide selection of usable materials, and the potential for diverse surface functionalizations.^[^ ^22^ ^]^ For example, poly(styrene)/ poly(urethane) nanofibers were modified with poly(ethylene imine) and triethylamine by impregnation to increase the CO_2_ adsorption capability. By optimization, 1.64 mmol g^−1^ CO_2_ could be adsorbed at 40 °C.^[^ ^23^ ^]^

Conversion of CO_2_ was shown by Hu et al. using Ni nanoparticles in mesoporous nanofibers for methanation of CO_2_.^[^ ^24^ ^]^ The corresponding electrochemical approaches were summarized by Zong et al.^[^ ^25^ ^]^

Poly(ethylene oxide) (PEO) is a CO_2_‐philic polymer. CO_2_ is soluble in PEO membranes which is caused by favorable quadrupole‐dipole interactions between CO_2_ and the ethylene oxide groups.^[^ ^26^, ^27^ ^]^ PEO was already used as a copolymer to increase CO_2_ diffusion through the CO_2_ separation membrane.^[^ ^28^ ^]^ It is electrospinnable and was used as a second polymer in blend solutions increasing the spinnability of various materials, e.g., chitosan for tissue engineering applications,^[^ ^29^ ^]^ or for phase change materials in thermal energy regulation in combination with poly(ethylene glycol).^[^ ^30^ ^]^ Since PEO is water soluble, it gives the opportunity of environmentally friendly electrospinning by using water as a green solvent.^[^ ^31^ ^]^

Branched poly(ethylene imine) (PEI) contains primary, secondary, and tertiary amines for CO_2_ capture. As a water‐soluble polymer containing amine groups for enhanced CO_2_ capturing it was already used for poly(acrylic acid) membranes where PEI was added in a postmodification step.^[^ ^32^ ^]^ PEI was also introduced in porous SiO_2_‐ structures to introduce CO_2_ capturing ability.^[^ ^33^, ^34^ ^]^

Typically, CO₂ hydrogenation to methanol occurs between 200 and 300 °C, with most catalysts operating above 200 °C.^[^ ^35^, ^36^ ^]^ However, recent studies have revealed several catalytic systems capable of functioning at lower temperatures. Pt nanoparticles encapsulated within Zr‐based UiO‐67 metal–organic frameworks demonstrated increased selectivity for methanol at 170 °C. Pt single‐atom catalyst Pt_1_@MIL, an ensemble of Pt single atoms coordinated with oxygen atoms in MIL‐101, has shown activity at a low temperature of 150 °C.^[^ ^37^ ^]^ A MoS_2_ nanosheet catalyst was able to achieve methanol formation at 180 °C.^[^ ^38^ ^]^

Copper‐based catalysts are widely used for CO₂ reduction, with CO₂ hydrogenation to methanol offering potential as a fuel or platform chemical in a closed carbon cycle.^[^ ^10^, ^33^, ^34^, ^35^, ^36^, ^37^, ^38^ ^]^ While the industrial Cu/ZnO/Al₂O₃ catalyst, commonly used for methanol synthesis from syngas, also demonstrates effectiveness for CO₂ hydrogenation, various attempts have been performed to develop more effective copper‐based catalysts for this reaction.^[^ ^39^, ^40^, ^41^ ^]^ Only single‐atom copper catalysts have been found to be able to catalyze the reaction at temperatures below 200 °C. C_3_N_4_‐supported Cu single‐atom catalysts have exhibited activity at 150 °C.^[^ ^42^ ^]^ The single‐atom Cu–Zr catalyst with Cu_1_–O_3_ units contributes solely to methanol synthesis at ≈180 °C, while the presence of small copper clusters or nanoparticles results in CO formation.^[^ ^43^ ^]^ It was revealed that Cu single atoms on ZnO support catalyze CO_2_ hydrogenation to methanol in the presence of water at 170 °C.^[^ ^44^ ^]^

To increase the introduced functionalities in electrospun nanofibers, the electrospinning method can be modified. Side‐by‐side electrospinning can be used for the implementation of different functional groups in two parts of a fiber, resulting in close contact of the groups.^[^ ^17^, ^45^, ^46^ ^]^ By combining PEI and copper nanoparticles (CuNP) in side‐by‐side spun solutions, adsorption can take place at amine groups in one part of the fibers and conversion at the CuNP in the second part. With both functionalities integrated in the same material, the release of the CO_2_ is not necessary, but the captured CO_2_ can be converted directly.

In this work, we introduce a bifunctional nanofiber for carbon capture and conversion prepared via side‐by‐side electrospinning of a PEO/PEI blend solution and a PEO solution loaded with CuNP using water as a green solvent for both solutions. The nanofibers are analyzed by using scanning electron microscopy (SEM), thermogravimetric analysis (TGA), and differential scanning calorimetry (DSC). The thermal stability of the fibers, as well as their CO_2_ adsorption capacity and the surface area are investigated. All of this is done in comparison to the single parts of the nanofibers, namely nanofibers consisting of PEO/PEI only and PEO/CuNP only. The catalytic activity of the bifunctional nanofibers regarding a batch reaction of CO_2_ hydrogenation with H_2_ at 150 °C was examined.

## 2 Results and Discussion

**Figure 1:**
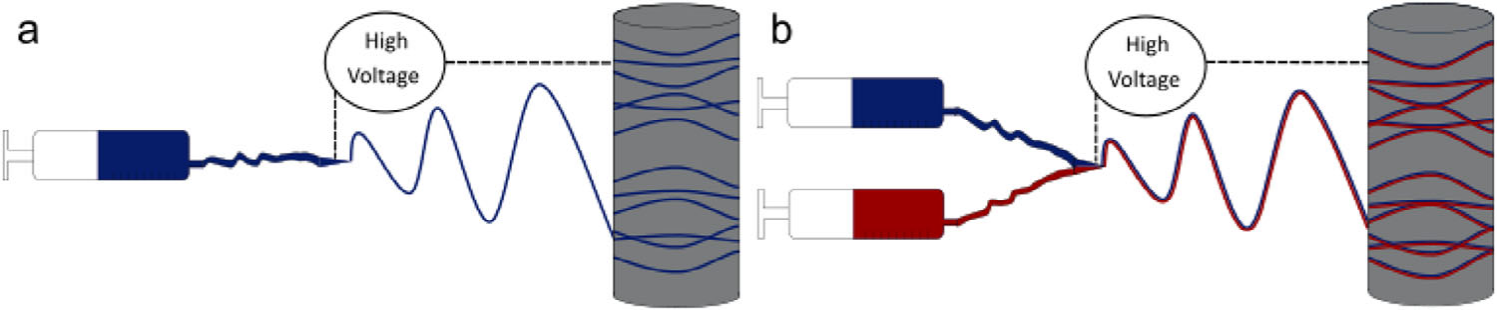
Schematic representation of electrospinning of one blend solution (a) and side‐by‐side spinning of two solutions (b).

Four different samples were produced via electrospinning. Three of them were fabricated using blend electrospinning, which were pristine PEO/PEI nanofibers, a sample of PEO mixed with CuNP named PEO/CuNP in the following, and one sample of PEO mixed with CuNP and blended with PEI which will be called PEO/PEI/CuNP. Additionally, PEO/PEI was spun side‐by‐side with PEO/CuNP. These fibers will be called PEO/PEI||PEO/CuNP. The blend electrospinning as well as the side‐by‐side electrospinning are shown schematically in **Figure** **1**.

**Figure 2:**
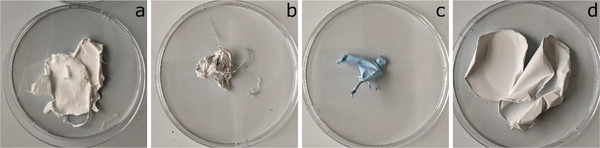
Electrospun nanofibers made from PEO/PEI (a), PEO/CuNP (b), PEO/PEI/CuNP (c) PEO/PEI||PEO/CuNP (d).

Electrospun pristine PEO/PEI fibers were collected as white homogenous mats (**Figure** **2**a). By electrospinning PEO with CuNP, a grey mat was produced from the black solution (Figure 2b). By adding PEI to the initial black solution of PEO and CuNP, it turned blue overnight, indicating an oxidation of the copper and the formation of Cu‐amine complexes.^[^ ^47^ ^]^ The resulting electrospun nanofibrous mat was also blue (Figure 2c) and not further analyzed. Via side‐by‐side electrospinning of PEO/PEI blend solution and PEO/CuNP solution a homogenously grey mat could be produced (Figure 2d), indicating the incorporation of the unaltered CuNP.

**Figure 3:**
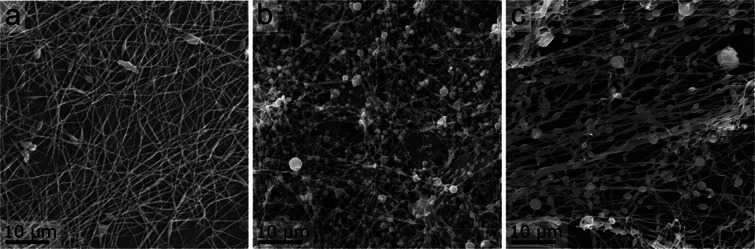
SEM micrographs of PEO/PEI fibers (a), PEO/CuNP fibers (b) and PEO/PEI||PEO/CuNP fibers (c).

SEM micrographs of the electrospun fibers (**Figure** **3**) show an almost homogeneous morphology of the pristine PEO/PEI fibers with few beads and a highly beaded morphology of the PEO/CuNP fibers as well as the side‐by‐side electrospun PEO/PEI||PEO/CuNP hybrid fibers. PEO/CuNP fibers are significantly thinner than PEO/PEI fibers. PEO/PEI||PEO/CuNP can be regarded as a combination of the thickness of PEO/PEI and the beaded structure of PEO/CuNP. As already discussed in the literature, the beads on a string morphology is not necessarily a disadvantage from an application point of view. The beads on a string morphology might be an advantage since it leads to an increased number of cavities, a lower pressure drop, and an increased surface area.^[^ ^48^, ^49^, ^50^ ^]^

**Figure 4:**
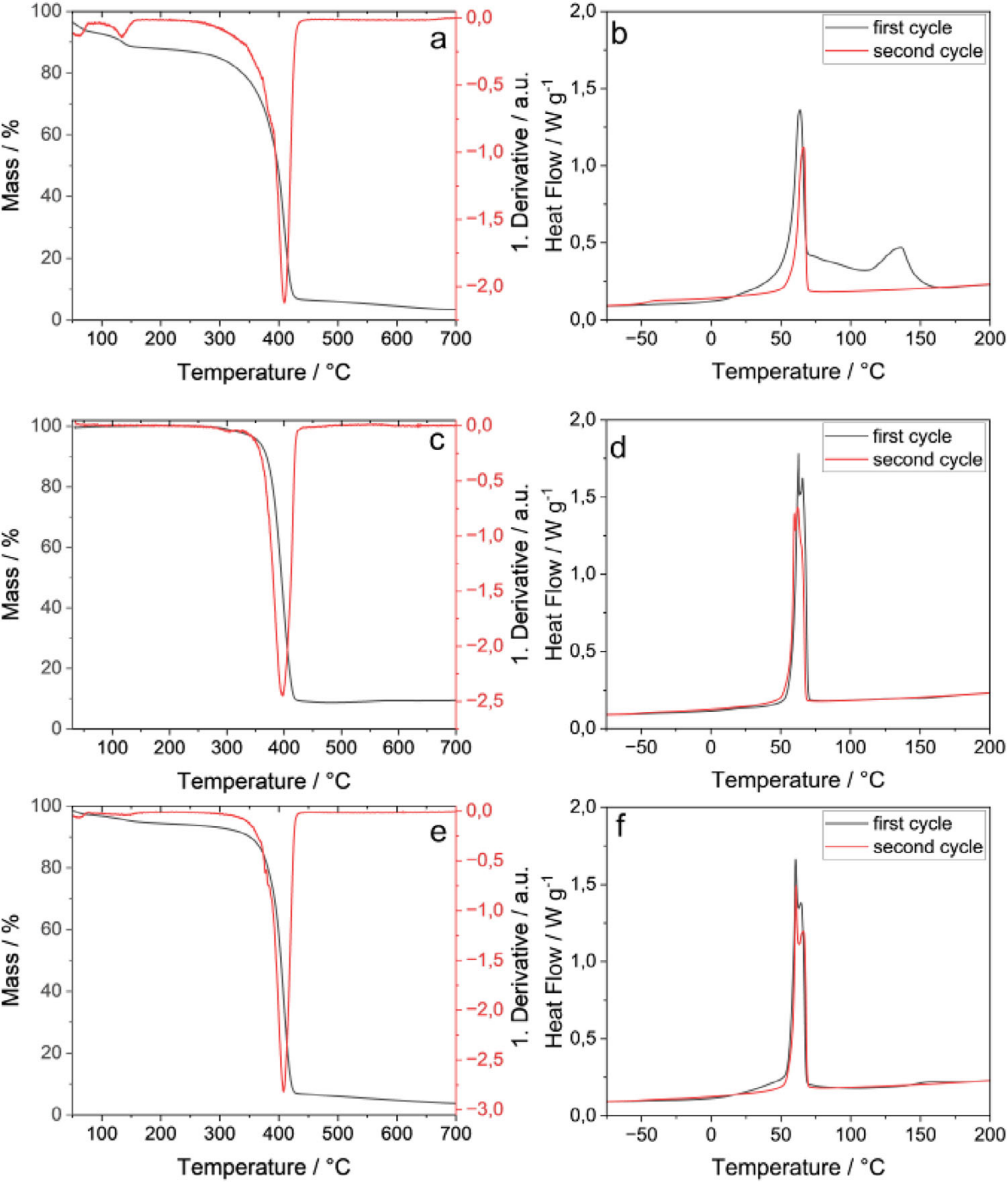
Thermogravimetric analysis with corresponding derivative (a,c,e) and differential scanning calorimetry (b,d,f) of PEO/PEI fibers (a,b), PEO/CuNP fibers (c,d) and PEO/PEI||PEO/CuNP fibers (e,f).

The three different fibers were examined using TGA and DSC. Slight deviations in thermal properties were observed (**Figure** **4** and **Table** **1**). For PEO/PEI fibers, a weight loss below the onset temperature of 380 °C can be explained by adsorbed water being evaporated (Figure 4a). 3.4% of the mass remains after heating up to 700 °C. For the PEO/CuNP there is no significant water evaporation, and the onset temperature is 373 °C (Figure 4c). The higher remaining mass of 9.5% can be explained by the added CuNP which do not degrade at these temperatures. Here, it should be taken into account, that 10 wt% of CuNP were added, indicating a higher degradation of polymer compared to the pristine fibers. For the side‐by‐side electrospun PEO/PEI||PEO/CuNP fibers the evaporation of adsorbed water below the onset temperature can be seen again (Figure 4e). The onset temperature is 388 °C, the highest of all three samples. The remaining mass is 3.5 % and comparable to the one of the pristine fibers. This indicates a higher loss of polymers compared to PEO/PEI since there are 5 wt% CuNP incorporated.

**Table 1:** Onset temperature (T_Onset_) of thermal degradation, remaining masses after heating up to 700 °C, and transition temperatures of PEO/PEI, PEO/CuNP, and PEO/PEI||PEO/CuNP.

	PEO/PEI	PEO/CuNP	PEO/PEI||PEO/CuNP
T _Onset / °C_	380	373	388
Mass _700 °C / %_	3.4	9.5	3.5
T _Peak, first cycle / °C_	63.5 135.6	62.8 / 65.4	60.5 / 64.4 155.0
T _Peak, second cycle / °C_	66.2	59.8 / 62.2	60.7 / 66.0
T_g / °C_	−47	−50	−49

For the first heating cycle in DSC measurements, two transition temperatures are observed for PEO/PEI fibers (Figure 4b) and for PEO/PEI||PEO/CuNP fibers (Figure 4f). The first transition temperature ≈60 to 64 °C corresponds to the melting temperature (T_m_) of PEO.^[^ ^29^ ^]^ The melting temperature of pure PEO without further processing was determined at 68 °C in the first cycle and 65 °C in the second heating cycle (Figure S1 and Table S1, Supporting Information). For PEO/PEI a second transition temperature at 135 °C can be seen which is shifted to 155 °C in the side‐by‐side electrospun hybrid fibers and corresponds to the evaporation of volatile components. A magnification of the second peak in PEO/PEI||PEO/CuNP fibers is shown in the (Figure S2, Supporting Information). For pure PEI, no melting temperature could be observed for the examined temperature range but evaporation of volatile components was observable (Figure S1 and Table S1, Supporting Information). For the PEO/CuNP fibers, the melting peak of PEO can be observed with two separated maxima at 63 and 65 °C (Figure 4d). This can be explained by a loosely packed sample in the pan which allows movement of the sample accompanied by differences in contact between sample and pan. The same effect can be observed for PEO/PEI||PEO/CuNP fibers, as well.

In the second heating cycle, only one transition can be observed at ≈60 to 66 °C for all three samples. The glass transition temperature (T_g_) can be observed between −47 and −50 °C. The cooling cycles of all samples are shown in (Figure S3, Supporting Information).

**Figure 5:**
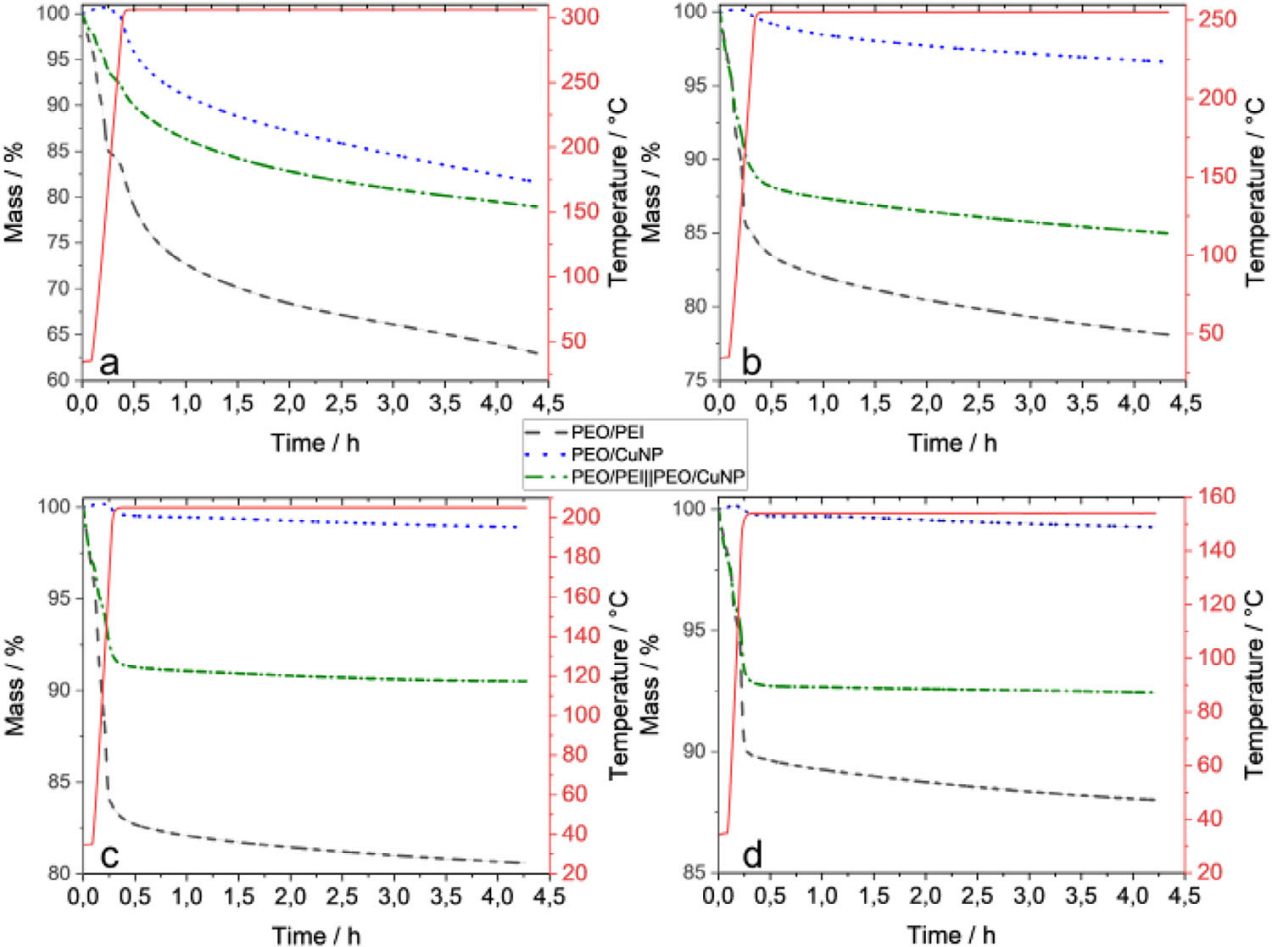
Mass of PEO/PEI (black), PEO/CuNP (blue), and PEO/PEI||PEO/CuNP (green) fibers while heating at 300 °C (a), 250 °C (b), 200 °C (c) and 150 °C (d) for 4 h with corresponding heating curves (red).

**Figure 6:**
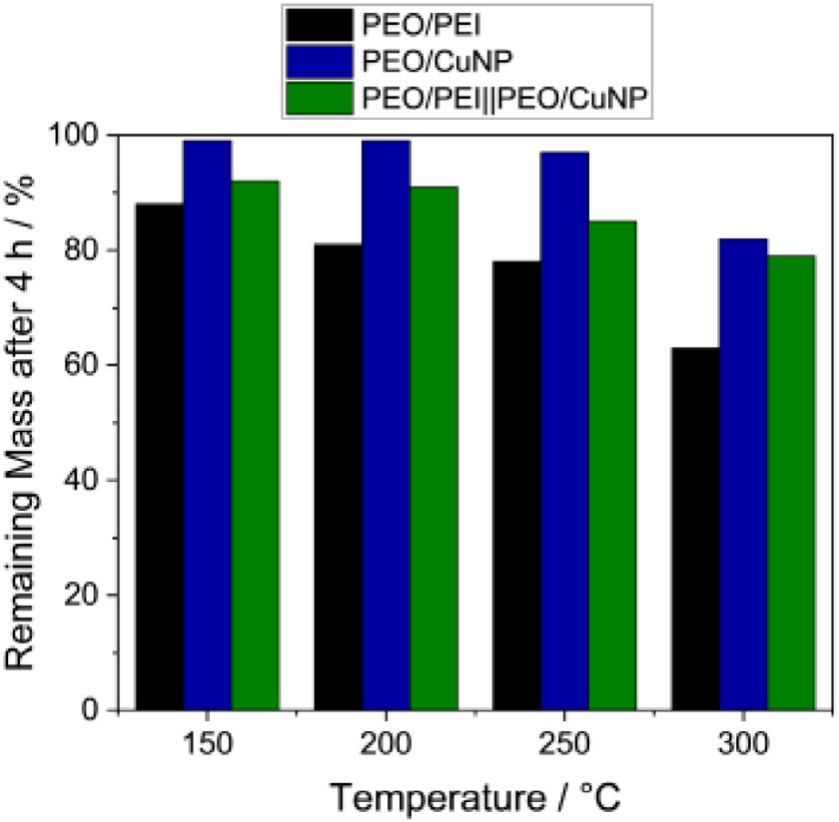
Remaining Mass of PEO/PEI (black), PEO/CuNP (blue), and PEO/PEI||PEO/CuNP (green) fibers after heating at 150, 200, 250, and 300 °C for 4 h.

The catalytic conversion of CO_2_ has been performed at elevated temperatures. Therefore, the thermal stability of the fibers is one of the key parameters and was investigated by adjusted TGA measurements as well. Here, the heating was done fast but the samples were kept at high temperatures for longer times (4 h) to monitor the weight loss allowing to follow the degradation of the polymers at a given temperature (**Figure** **5**). For all temperatures, the PEO/CuNP fibers are the most stable, and PEO/PEI fibers are losing the most weight. The thermal stability of the side‐by‐side electrospun PEO/PEI||PEO/CuNP fibers is between the single‐component fibers. At 300 °C the remaining masses are 82, 63, and 79% for PEO/CuNP, PEO/PEI, and PEO/PEI||PEO/CuNP, respectively (Figure 5a). With lower temperatures, the samples are less degraded. For the side‐by‐side electrospun fibers 85, 91, and 92% of the used sample remain after heating for 4 h at 250, 200, and 150 °C. At 150 °C 88% of the PEO/PEI fibers and 99% of the PEO/CuNP fibers remained after the heating experiment. A sufficiently high thermal stability at 150 °C can be concluded (Figure 5d). The remaining masses after heating at a certain temperature for 4 h are visualized in **Figure** **6**.

**Figure 7:**
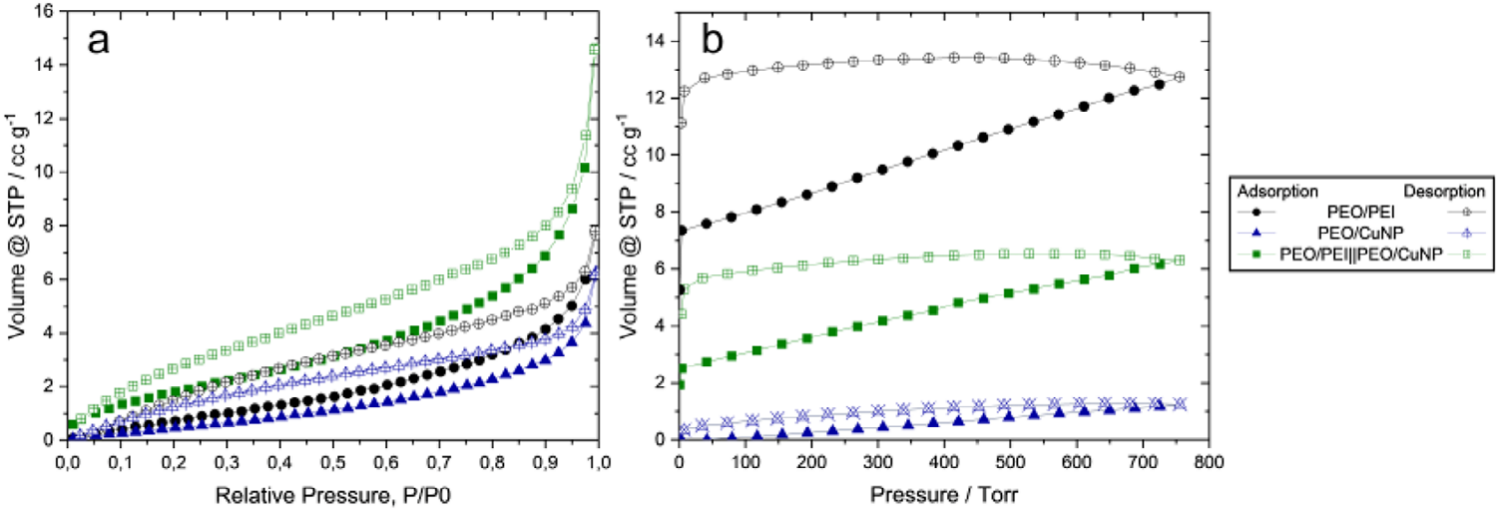
Nitrogen adsorption (filled symbols) and desorption (hollow symbols) of PEO/PEI (black), PEO/CuNP (blue) and PEO/PEI||PEO/CuNP (green) (a) and CO_2_ adsorption (filled symbols) and desorption (hollow symbols) of PEO/PEI (black), PEO/CuNP (blue) and PEO/PEI||PEO/CuNP (green) (b).

**Figure** **7** presents the nitrogen and CO_2_ adsorption‐desorption isotherms. The surface area is highest for PEO/PEI||PEO/CuNP fibers with 7 m^2^ g^−1^ and lowest for PEO/CuNP with 3 m^2^ g^−1^. For PEO/PEI fibers the surface area is 5 m^2^ g^−1^. A direct correlation to the CO_2_ adsorption of the samples can not be seen (**Table** **2**). Here, PEO/PEI performs best with 0.56 mmol g^−1^. PEO/PEI||PEO/CuNP shows CO_2_ adsorption of 0.28 mmol g^−1^ and for PEO/CuNP almost no adsorption could be measured with 0.06 mmol g^−1^. Here, the content of amines in the samples needs to be considered. Neither PEO nor CuNP are known to adsorb high amounts of CO_2_. Adding amine‐containing PEI to PEO leads to significantly higher amounts of adsorbed CO_2_. The combination of PEO/CuNP with low adsorption performance and PEO/PEI with higher performance leads to a material combining both and showing almost averaged adsorption.

**Table 2:** CO_2_‐adsorption and surface area of PEO/PEI, PEO/CuNP, and PEO/PEI||PEO/CuNP.

Sample	CO_2_ adsorption / mmol g^−1^	Surface area / m^2^ g^−1^
PEO/PEI	0.56	5
PEO/CuNP	0.06	3
PEO/PEI||PEO/CuNP	0.28	7

Optimization of the CO_2_ adsorption might either be done by increasing the surface area for example, by introducing porosity to the fibers,^[^ ^50^, ^51^, ^52^ ^]^ by synthesis of less dense 3D fibrous networks,^[^ ^53^, ^54^ ^]^ or by incorporation of higher amounts of amines. Regarding the amount of introduced amines, it needs to be considered that our preparation method leads to partially covered functional groups since the PEI is not exclusively on the surface of the fibers. By postmodification via impregnation with PEI CO_2_ adsorption between 0.48 and 1.64 mmol g^−1^ at 40 °C was shown.^[^ ^23^ ^]^ Here, the functional groups are located only on the surface but it needs to be considered that this might lead to a coverage of additional functional groups as well as a decrease in surface area. The surface areas of coated materials were found from 23.56 m^2^ g^−1^ down to 4.05 m^2^ g^−1^ compared to 62.27 m^2^ g^−1^ for the uncoated material.^[^ ^23^ ^]^

CO_2_ hydrogenation was performed over PEO/PEI||PEO/CuNP nanofibers. The blanc experiment of CO_2_ hydrogenation with an empty reactor shows no CO_2_ transformation. The catalytic experiment with PEO/PEI sample shows that the resulting gas mixture presented moisture. The CO_2_ to H_2_ ratio was 1:4, the same as in the starting reaction mixture. Considering the mass loss of PEO/PEI after treatment at 150 °C it can be concluded that there are no catalytic transformations occurring, whereas the water is emitted from PEO/PEI.

Results of CO_2_ hydrogenation using PEO/PEI||PEO/CuNP nanofibers show methanol formation during the catalytic process. No other products of CO_2_ hydrogenation were determined in the reaction products. At a low temperature of 150 °C a conversion of 0.3% was achieved with a space time yield (STY) of 0.35 g kg^−1 ^h^−1^. This result shows a 100% selectivity toward methanol for the presented conditions. The obtained result indicates that the main part of CuNP is covered by polymer. This conclusion is supported by a comparative analysis of the productivity of Cu catalysts reported in the literature. The typical turnover number (TON) for Cu surface is 1.6·10^−2^ molecule CH_3_OH s^−1^ site^−1^.^[^ ^55^, ^56^ ^]^ Based on approximations that all surface Cu atoms are active and the specific activity of Cu is constant, obtained TON for PEO/PEI||PEO/CuNP, is 1.5·10^−5^ molecule CH_3_OH s^−1^ site^−1^, which indicates that the catalytically active surface of Cu is 0.1% of the total Cu surface. Therefore, the first principle fabrication of catalytically active polymer‐Cu bifunctional composites is shown. It can be proposed that the creation of composites with high productivity can be achieved by modification of the synthetic procedure with an increase of active surface Cu.

Our catalytic study shows that not only Cu single‐atom catalysts^[^ ^42^, ^43^, ^44^ ^]^ but also catalysts containing CuNP are able to catalyze CO_2_ hydrogenation to methanol at temperatures below 200 °C. Moreover, contrary to the data reported recently that copper nanoparticles support CO formation, our results indicate a possibility of reaching 100% selectivity for methanol. Perhaps, the direction of CuNP action either toward CO or methanol formation is strongly dependable on the effect of support. Compared to the data presented in the literature for copper catalysts, the activity of the reported system is pretty low. However, it shows that even using commercial CuNP without further modification added to a polymer matrix, which might even cover part of the particles, results in the fabrication of a catalyst for CO_2_ to methanol conversion at low temperatures. Therefore, there is a window for a proper design of integrated CCU systems based on CuNP and polymer matrix, which operates at low temperatures for CO_2_ transformation to methanol. Further progress may be reached by a proper modification of the system, e.g., using bimetallic catalysts,^[^ ^57^, ^58^, ^59^ ^]^ involving ZnO,^[^ ^44^, ^60^ ^]^ CeO_2_,^[^ ^61^ ^]^ and/or water,^[^ ^44^ ^]^ or modification of a catalyst with ZnO, ZrO_2_, and MgO.^[^ ^62^, ^63^ ^]^ That will require optimization of the catalytic properties as well as the CO_2_ adsorption, trying to explore the whole potential of bifunctional fibers with independently adjustable functions.

## 3 Conclusion

Bifunctional electrospun polymer nanofibers for CO_2_ capture and conversion were fabricated using side‐by‐side electrospinning. Water‐based PEO/PEI blend solution and PEO/CuNP were used and the resulting fibers show combined properties of both components. For comparison of material properties, the solutions were also electrospun independently by simple blend electrospinning. Characterization of fibers by SEM, TGA, and DSC verifies their combined characteristics. Investigations of fibers ability to adsorb CO_2_ and catalytic studies show promising results for CCU technology. The introduced imines are able to capture CO_2_ and, without the necessity of release, hydrogenation to methanol can be done at commercial CuNP without further modification at a low temperature of 150 °C. Both functionalities are incorporated into the side‐by‐side electrospun hybrid nanofibers with a CO_2_ adsorption of 0.28 mmol g^−1^ and a STY for the reaction to methanol of 0.35 g kg^−1 ^h^−1^. Here, it should be considered that both functionalities are not optimized yet but combined in one material, which leads to shorter diffusion paths and lower energy consumption since no release of captured CO_2_ is necessary.

Optimization of both functions is needed in the future but can be done independently by modification of fiber morphology, incorporation of higher amounts of amines, or variations of incorporated catalyst. Therefore, the introduced bifunctional electrospun hybrid nanofibers represent a promising new class of materials, demonstrating the potential for further optimization of similar systems.

## 4 Experimental Section

### Materials

The chemicals were bought from Sigma Aldrich, Merck KGaA, Darmstadt, Germany, and used without further purification. Poly(ethylene oxide) (PEO, M_v_ = 300 000 g mol^−1^) was used as the spinnable polymer matrix. Poly(ethylene imine) (PEI, branched, 50% in H_2_O, M_n_ = 60 000 g mol^−1^ determined by gel permeation chromatography, M_w_ = 750 000 g mol^−1^ determined by light scattering) was used as a source for amines and commercial copper nanoparticles (CuNP, 25 nm particle size determined by TEM) were used as a catalyst.

### Methods—Preparation of Electrospinning Solutions

PEO and PEI were solved in water (6 wt%, each). For the polymer blend solution, the polymer solutions were mixed together (PEO/PEI 70/30 v/v). Additionally, the PEO solution was mixed with CuNP (10 wt% regarding to polymer weight). For blend electrospinning of all components, PEI was added to the PEO solution with CuNP (30/70 v/v). For side‐by‐side electrospinning, the separated PEO solution with CuNP and PEO/PEI solution were used. In all cases solving and mixing were performed by stirring at room temperature.

### Methods—Electrospinning

Electrospinning was done by applying a positive voltage of 17 kV to the polymer solution and −1 kV to the collector. The distance to the collector was 18 cm. The solutions were pumped with syringe pumps. In the case of blend spinning the flow rate was 0.3 mL h^−1^ and in the case of side‐by‐side spinning it was 0.15 mL h^−1^ for each syringe.

For side‐by‐side electrospinning, the needles of each solution were connected to each other with a pipette tip to bring the solutions in contact right before Taylor cone formation.

### Methods—Electron Microscopy

Scanning electron microscopy (SEM) was performed using a JSM‐IT 100 InTouchScope. The fibers were prepared by sputtering a thin gold coating using a Cressington Sputter Coater 108 auto to get appropriate conductivity.

### Methods—Thermal Analysis

Thermogravimetric analysis (TGA) was done with 10 mg of fibers in a ceramic crucible at a Perkin‐Elmer TGA 4000 system. The samples were kept at 30 °C for 5 min followed by heating up to 700 °C at 5 °C min^−1^ and kept at 700 °C for another 5 min.

For examination of thermal stability, the samples were kept at 30 °C for 5 min, heated at 150, 200, 250, or 300 °C at 15 °C min^−1,^ and kept at the high temperature for 4 h.

Differential scanning calorimetry (DSC) was done in Tzero‐Al‐hermetic pans with perforated lids. The measurements were done at a DSC Q2000 / TA Instruments system. Samples were equilibrated at −90 °C for 5 min and heated from −90 to 200 °C with 5 °C min^−1^. The temperature was kept for 5 min followed by cooling to −65 °C with 5 °C min^−1^. After keeping the temperature for another 5 min a second heating cycle up to 200 °C with 5 °C min^−1^ was performed.

### Methods—Gas‐Adsorption Measurements

The samples were dried under vacuum at room temperature for three days and degassed for another 24 h at 30 °C under 5 × 10^−10^ mbar vacuum. Measurements were performed at Autosorb iQ MP von Quantachrome. N_2_ sorption was measured at 77 K and CO_2_ sorption at 273 K. The removal time for helium was 15 min. ≈100 mg fibers were used for each measurement and adsorption as well as desorption were measured.

### Methods—Catalytic Experiments

Catalytic experiments were provided in a high‐pressure batch reactor with a volume of 150 mL at a total pressure of 50 bar and a temperature of 150 °С for 24 h. The mass of fibers was 500 mg, reaction was performed with 20% of CO_2_ (10 bar) and 80% of H_2_ (40 bar). The analysis of the composition of the reaction products gas mixture was performed using 2 different gas chromatographs with a thermal conductivity detector (TCD) and flame‐ionization detector (FID). A gas chromatograph with TCD was used for the determination of the concentration of carbon monoxide, hydrogen, CO_2_, formic acid, and formaldehyde, whereas a gas chromatograph with FID was used for the determination of the concentration of alcohols and hydrocarbons.

## Conflict of Interest

The authors declare no conflict of interest.

## Supporting information



Supporting Information

## Data Availability

The data that support the findings of this study are available from the corresponding author upon reasonable request.
