# Supplementary material for: Sustainable Bifunctional Electrospun Hybrid Nanofibers for CO2 Capture and Conversion
Source: Macromol Rapid Commun. 2025 May 27;46(14):2500050. doi: 10.1002/marc.202500050 (PMC12272532; doi:10.1002/marc.202500050)
Supplement: Supplementary file 1 — Supporting Information [file MARC-46-2500050-s001.docx]

# Supporting Information

# Sustainable Bifunctional Electrospun Hybrid Nanofibers for CO_2_ Capture and Conversion

R. Hengsbach,^1^ I. Bychko,^2^ S. Schwarz,^3^ P. Strizhak,^2^ A. Fahmi^1^

^1^ Faculty of Technology and Bionics, Rhine-Waal University of Applied Science, Marie-Curie-Straße 1, 47533 Kleve, Germany.

^2^ L. V. Pisarzhevskii Institute of Physical Chemistry, National Academy of Sciences of Ukraine, 31 Prosp. Nauky, 03028 Kyiv, Ukraine.

^3^ Leibniz-Institut für Polymerforschung Dresden e. V., Hohe Straße 6, 01069 Dresden, Germany.

## DSC Measurements

As references, pure PEO and PEI were examined via differential scanning calorimetry. PEO was used as received. PEI was freeze-dried before measurement. Measurements were done as described in the experimental part of the manuscript, except for the first heating cycle of PEO. PEO was heated only until 100 °C (Figure S1).


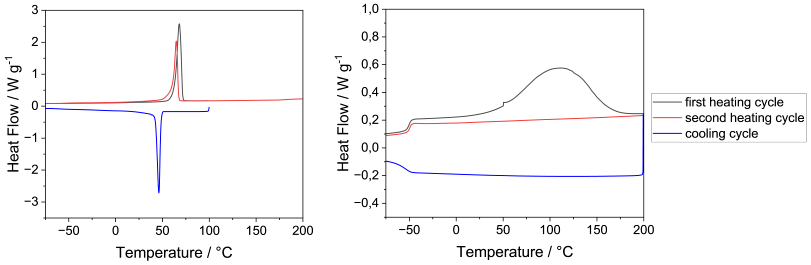


Figure S1: Differential scanning calorimetry of PEO (left) and PEI (right).

PEO has a melting temperature (T_m_) of 68 °C in the first heating cycle and 65 °C in the second cycle. For PEI, no melting temperature can be observed in the given temperature range. The broad peak in the first heating cycle can be explained as the evaporation of volatile compounds and is not present in the second cycle. The glass transition temperatures (T_g_) of PEO and PEI are -53 °C and -50 °C, respectively (Table S1).

Table S1: Transition temperatures of PEO and PEI.

|  | PEO | PEI |
| --- | --- | --- |
| T _m, first cycle_ / °C | 67.9 | - |
| T _m, second cycle_ / °C | 64.7 | - |
| T_g_ / °C | -52.5 | -50 |

For better visualization, the second peak occurring in DSC measurements of PEO/PEI||PEO/CuNP is magnified. The magnified region of the graph is highlighted (Figure S2).


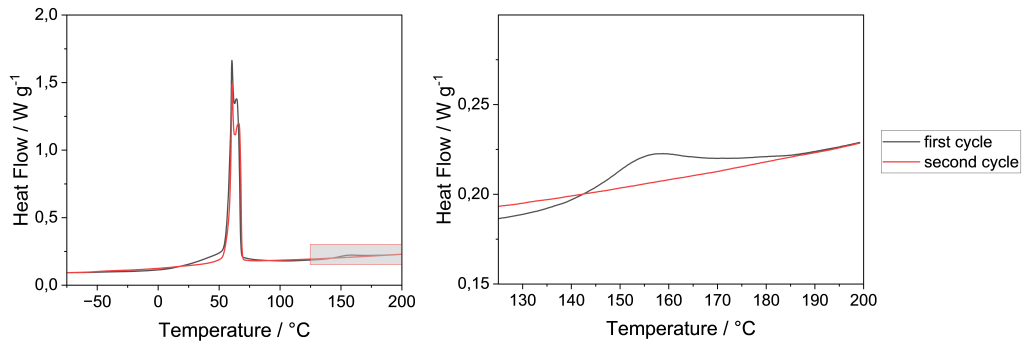


Figure S2: Differential scanning calorimetry of PEO/PEI||PEO/CuNP fibers (left) with a close-up view for the second peak (right).

The broad second peak with low intensity is assigned to the evaporation of volatile compounds.

Figure S3 presents the data of cooling cycles in between the first and second heating cycles of PEO/PEI fibers, PEO/CuNP fibers, and PEO/PEI||PEO/CuNP fibers.


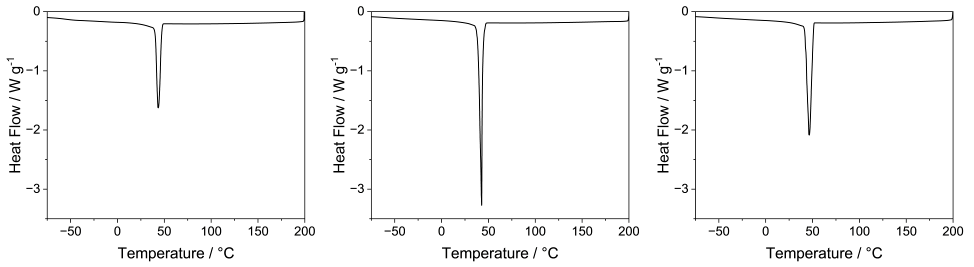


Figure S3: Differential scanning calorimetry cooling cycles of PEO/PEI fibers (left), PEO/CuNP fibers (middle) and PEO/PEI||PEO/CuNP fibers (right).

The peak minima for PEO/PEI fibers, PEO/CuNP fibers, and PEO/PEI||PEO/CuNP are observed at 43.5 °C, 42.8 °C, and 46.5 °C, respectively.
